# Theoretical Study of N-Heterocyclic-Carbene–ZnX_2_ (X = H, Me, Et) Complexes

**DOI:** 10.3390/ma14206147

**Published:** 2021-10-16

**Authors:** Mirosław Jabłoński

**Affiliations:** Faculty of Chemistry, Nicolaus Copernicus University in Toruń, Gagarina 7, 87-100 Toruń, Poland; teojab@chem.umk.pl; Tel.: +48-056-611-4695

**Keywords:** carbene, N-heterocyclic carbene, NHC, zinc, zinc bond, organometallic chemistry, steric effects, spatial hindrance, intermolecular interaction, DFT

## Abstract

This article discusses the properties of as many as 30 carbene–ZnX${}_{2}$ (X = H, Me, Et) complexes featuring a zinc bond C⋯Zn. The group of carbenes is represented by imidazol-2-ylidene and its nine derivatives (labeled as IR), in which both hydrogen atoms of N-H bonds have been substituted by R groups with various spatial hindrances, from the smallest Me, ${}^{i}$Pr, ${}^{t}$Bu through Ph, Tol, and Xyl to the bulkiest Mes, Dipp, and Ad. The main goal is to study the relationship between type and size of R and X and both the strength of C⋯Zn and the torsional angle of the ZnX${}_{2}$ plane with respect to the plane of the imidazol-2-ylidene ring. Despite the considerable diversity of R and X, the range of $d_{C\cdots \mathrm{Zn}}$ is quite narrow: 2.12–2.20 Å. On the contrary, $D_{0}$ is characterized by a fairly wide range of 18.5–27.4 kcal/mol. For the smallest carbenes, the ZnX${}_{2}$ molecule is either in the plane of the carbene or is only slightly twisted with respect to it. The twist angle becomes larger and more varied with the bulkier R. However, the value of this angle is not easy to predict because it results not only from the presence of steric effects but also from the possible presence of various interatomic interactions, such as dihydrogen bonds, tetrel bonds, agostic bonds, and hydrogen bonds. It has been shown that at least some of these interactions may have a non-negligible influence on the structure of the IR–ZnX2 complex. This fact should be taken into account in addition to the commonly discussed R⋯X steric repulsion.

## 1. Introduction

**Figure 1:**
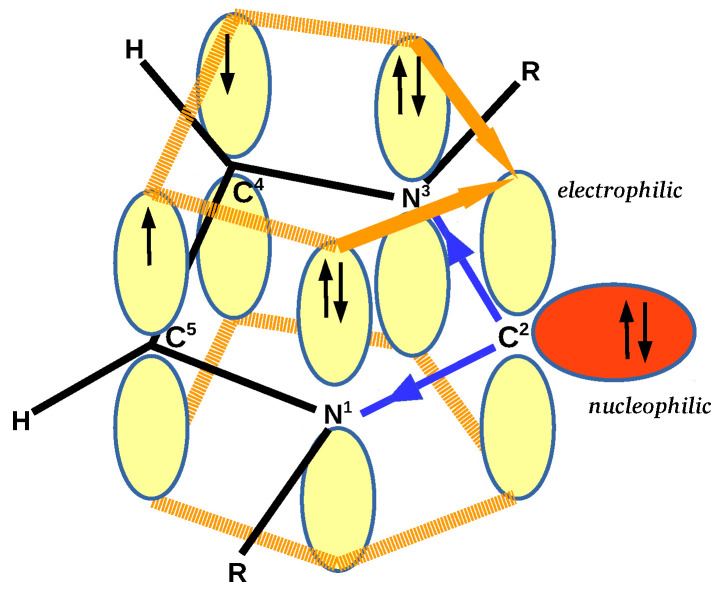
Electronic structure of R-substituted imidazol-2-ylidene.

Undoubtedly, carbenes belong to one of the most important groups of compounds in both organic and organometallic chemistry [1,2,3,4,5,6,7,8,9,10,11,12,13,14,15,16,17,18]. Among them, the so-called N-heterocyclic carbenes (NHCs), especially those derived from imidazol-2-ylidene, occupy a superior position [4,8,9,11,12,15,16,17,18]. This, in turn, results from their unique electronic structure (Figure 1).

In the singlet state, the divalent carbene carbon atom has a lone electron pair in the ring plane and an unoccupied *p*-orbital perpendicular to this plane. Importantly, this singlet state is stabilized by the simultaneous presence of several important factors. First of all, the presence of nearby nitrogen atoms is crucial, which stabilize the singlet state through both the inductive σ-electron withdrawing effect (indicated by the blue arrows in Figure 1) and the π-electron donation from lone electron pairs to the empty *p*-orbital of the C${}^{2}$ carbon atom (orange arrows) [19,20,21,22,23]. Moreover, another advantageous factor is the π-electron delocalization throughout the ring (imparting an aromatic character) [24] and a small N1-C2-N3 angle [19,25,26]. Importantly, while all these stabilizing factors are present in imidazol-2-ylidene, they do not need to coexist in other NHCs [16]. A very important factor affecting the stabilization of NHCs is the size of the R substituent attached to nitrogen atoms. In general, larger substituents increase the stabilization of NHC by sterically preventing carbene dimerization [27].

The simultaneous presence of a lone electron pair and the formally empty *p* orbital on the carbene carbon atom can cause, depending on the situation, the carbene molecule to exhibit its electrophilic [7,28,29,30,31,32,33] or nucleophilic properties, while the nucleophilic properties of NHCs are much more often explored in organic and organometallic synthesis and homogeneous catalysis [4,8,9,15,16,17,18]. In fact, the carbene lone electron pair can be donated to almost all elements of the periodic table. Among them, connections with transition metals, leading to NHC-M complexes, are the most important [8,12,17].

The enormous variety of NHC-M systems makes NHCs critical in organometallic catalysis [16,34,35,36,37,38]. For example, so-called Grubbs ruthenium-based catalysts are used in olefin metathesis, and NHC-Pd complexes are used in cross-coupling reactions. As for the former, the second-generation Grubbs catalysts in particular are widely used due to their high resistance to moisture and air. These catalysts can have both unsaturated and saturated heterocyclic rings. Examples include 1,3-bis(2,4,6-trimethylphenyl)imidazole [39,40,41] or 1,3-bis(2,4,6-trimethylphenyl)dihydroimidazole [42]. The second-generation catalysts are commercially available. The very popular Hoveyda–Grubbs catalysts [43] are derivatives of Grubbs catalysts. NHC-M complexes are also the basis of the third-generation Gibbs catalysts, the so-called fast-initiating catalysts [44]. They are most commonly used as initiators for ring opening metathesis polymerization (ROMP) [45]. Another equally important area of application of NHC-M complexes is material chemistry [46]. Some of them act as electrical conductors, phosphors, and other photoactive materials [47]. There is also growing interest in the medicinal applications of NHC-M complexes, especially those based on Ag(I) and Au (I) [48,49]. For example, many Ag(I) compounds with imidazol-2-ylidene exhibit antibacterial properties, while similar Au(I) compounds are promising anticancer drugs. In this case, the tuning of the appropriate substituents on the nitrogen is crucial, as their size has a major influence on the penetration through the mitochondrial membrane.

In the case of the NHC-M complexes, those involving zinc (i.e., M = Zn) are of increasing practical importance [35,36,38,50,51,52,53,54,55,56,57,58,59,60,61,62,63,64], due in part to the easy availability and relatively low cost of precursors of such complexes. Practical applications of NHC-Zn complexes in organometallic catalysis have recently been extensively described [34,35,36,38]. Among them, noteworthy is the zinc-catalyzed reduction (especially hydrosilylation) of unsaturated small molecules, e.g., CO${}_{2}$, carbonyls, alkenes, alkynes, imines, and nitriles. As already mentioned, the stabilization of the NHC-M complexes is greatly enhanced by the use of large R substituents attached to the nitrogens of the imidazole-2-ylidene ring. The most common bulky groups are mesityl (Mes), 2,6-diisopropylphenyl (Dipp), and 1-adamantyl (Ad). Definitely smaller, but also giving considerable sterical hindrance, are the *tert*-butyl (${}^{t}$Bu) and isopropyl (${}^{i}$Pr) groups. Although, for understandable reasons, such systems are the most attractive for experimental research and practical applications, they are troublesome for accurate theoretical studies due to a high computational cost for such large systems. It is enough to mention that the computational cost of the most common DFT method scales formally with respect to the number of basis functions as $N^{4}$ [65]. For this reason, theoretical computations are usually limited to the simplest NHCs (or other even smaller carbenes), i.e., imidazol-2-ylidene.

The aim of this article is to use computational chemistry methods to describe 30 complexes of the NHC–ZnX${}_{2}$ (X = Et, Me, H) type, where NHC is either imidazol-2-ylidene or its derivative, in which both hydrogen atoms from N-H bonds have been replaced by substituents representing various steric hindrances, from the smallest methyl (Me), isopropyl (${}^{i}$Pr), and *tert*-butyl (${}^{t}$Bu) through phenyl (Ph), 4-methylphenyl (Tol), and 2,6-dimethylphenyl (Xyl) to the bulkiest mesityl (Mes), 2,6-diisopropylphenyl (Dipp), and adamantyl (Ad). It should be emphasized that a large number of the considered complexes are also the subject of experimental research, as these complexes are an important intermediate in organometallic syntheses and catalysis. Moreover, for some of them, even crystallographic structures were found (IAd–ZnEt${}_{2}$ (WEGHAR) [50], IDipp–ZnEt${}_{2}$ (DEDHUQ) [55], IDipp–ZnMe${}_{2}$ (YIDQIN) [59]). The main goal will be to study the relationship between the size and type of the R group (and X in ZnX${}_{2}$) and the NHC–ZnX${}_{2}$ (NHC = IR) complex structure, mainly the torsional angle of the ZnX${}_{2}$ unit plane relative to the imidazol-2-ylidene ring plane. Apart from this issue, the influence of R and X on the bond strength of C⋯Zn (this bond will be hereinafter referred to as a zinc bond [64]) will be another important topic of interest. Taking into account both the small number of theoretical studies of NHC–ZnX${}_{2}$ systems (especially with large R substituents) and the lack of in-depth research on the effect of the size of the R and X groups on the torsional angle, the results presented here are novel and contribute to the knowledge of carbene chemistry.

## 2. Theoretical Methods

Geometries of all the systems were fully optimized on the MN15/6-311+G(d) level of theory utilizing Gaussian 16 [66]. There were no imaginary frequencies showing that equilibrium structures were obtained each time. The MN15 [67] exchange-correlation functional of DFT [68,69,70] was chosen due to the fact that MN15 was one of the few functionals recommended for calculations involving transition metal atoms in an excellent review article on the assessment of 200 functionals by Mardirossian and Head-Gordon [71]. Electrostatic potential maps, QTAIM-based [72,73,74] calculations, and NCI-based [75,76] plots were obtained by means of the AIMAll program [77]. Atomic charges were calculated by the Hirshfeld method [78,79,80], as it has been shown that they are chemically sound [64,81,82].

## 3. Results and Discussion

### 3.1. Investigated Systems

**Figure 2:**
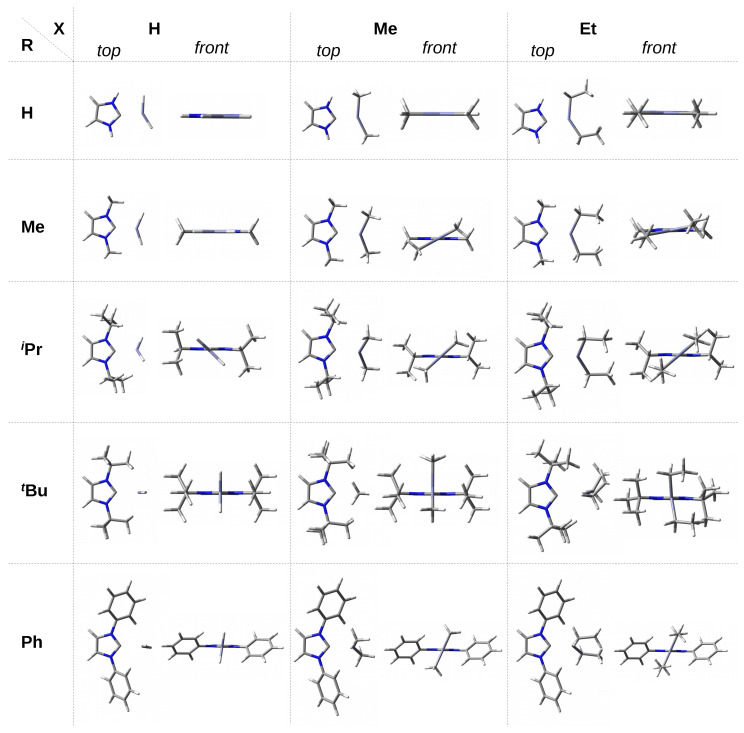
Top and front views of the fully optimized structures of the IR–ZnX${}_{2}$ (IR = double R-substituted imidazol-2-ylidene; R = H, Me, ${}^{i}$Pr, ${}^{t}$Bu, Ph; X = H, Me, Et) complexes.

**Figure 3:**
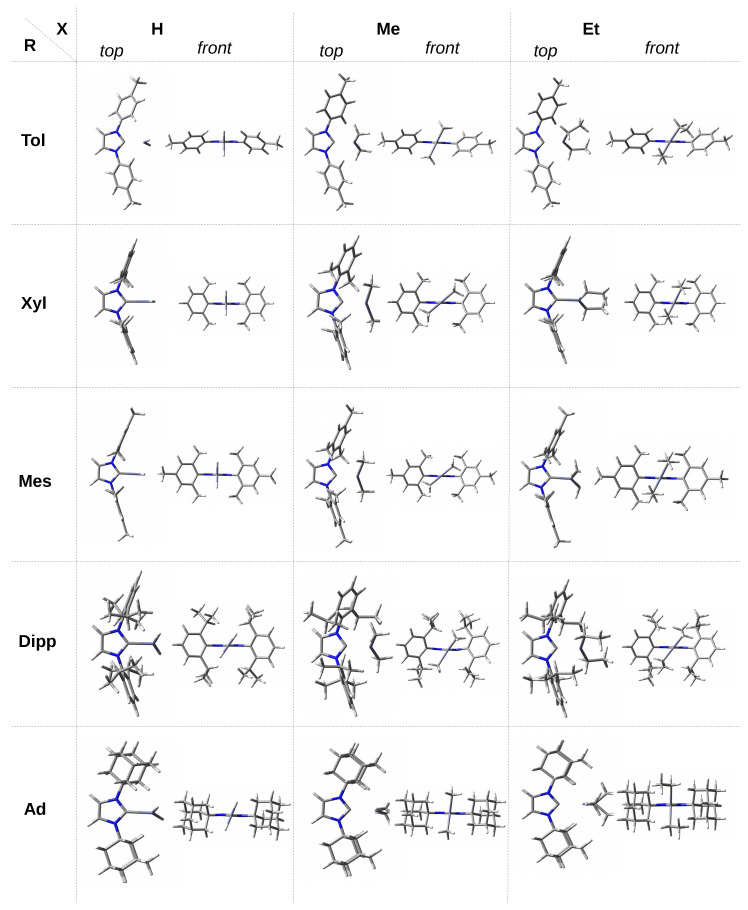
Top and front views of the fully optimized structures of the IR–ZnX${}_{2}$ (IR = double R-substituted imidazol-2-ylidene; R = Tol, Xyl, Mes, Dipp, Ad; X = H, Me, Et) complexes.

As already mentioned in the Section 1, 30 complexes of the NHC-ZnX${}_{2}$ type were considered, where X = H, Me, Et. The set of NHC carbenes consists of imidazol-2-ylidene and its nine derivatives, in which both hydrogen atoms from the N-H bonds have been replaced by groups leading to various steric hindrance at the C${}^{2}$ carbene carbon atom. These groups (R) are as follows: methyl (Me), isopropyl (${}^{i}$Pr), *tert*-butyl (${}^{t}$Bu), phenyl (Ph), 4-methylphenyl (Tol), 2,6-dimethylphenyl (Xyl), mesityl (Mes), 2,6-diisopropylphenyl (Dipp), and adamantyl (Ad). For convenience, individual complexes will be designed as IR-ZnX${}_{2}$. The structures obtained are shown in Figure 2 and Figure 3 for carbenes with the smaller and bulkier R substituents, respectively.

The front view of the obtained structures of the discussed IR–ZnX${}_{2}$ complexes allows one to immediately notice that the ZnX${}_{2}$ unit is positioned differently in relation to the plane of the carbene molecule ring. Namely, in the case of the smallest carbenes, i.e., I and IMe, the ZnX${}_{2}$ molecule is either in the same plane or only slightly twisted in relation to this plane. For larger R, however, the twist is considerable, and in some cases the ZnX${}_{2}$ molecule may even have a perpendicular orientation. Therefore, the alignment of the X substituents in the ZnX${}_{2}$ molecule with respect to the ring plane of the carbene is a very important issue and, in my opinion, not described frequently enough. For this reason, this issue will be an important topic of this article.

### 3.2. Geometric and Energetic Characteristics of the C⋯Zn Interaction

The most important parameters characterizing a given interaction are its length and its strength, where the strength can be described in various ways, e.g., by the dissociation energy. However, when it comes to the dissociation energy (or interaction energy), it actually concerns the entire dimer, i.e., it is a global quantity, and only when the described interaction is dominant can the determined dissociation energy describe this interaction [83]. The distances C⋯Zn and the dissociation energies determined for the discussed complexes are presented in Table 1 and Table 2, respectively.

It is somewhat surprising that despite such diverse R substituents and different X groups, the range of C⋯Zn distances is very narrow, from ca. 2.12 Å (IMes–ZnH${}_{2}$) to 2.20 Å (IPh–ZnMe${}_{2}$). On the contrary, the range of the dissociation energy values is quite wide (ca. 9 kcal/mol). The strongest C⋯Zn interaction (27.4 kcal/mol) is in agreement with the shortest C⋯Zn contact in IMes–ZnH${}_{2}$, and, conversely, the longest contact in IPh–ZnMe${}_{2}$ is characterized by the lowest dissociation energy (18.5 kcal/mol). However, the overall relationship between the length of C⋯Zn and the dissociation energy is weak if all the complexes are considered together.

**Figure 4:**
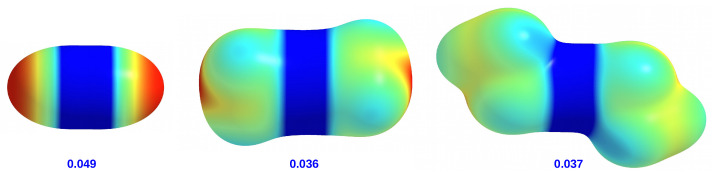
Maps of the total electrostatic potential projected on 0.001 au isodensity surfaces of ZnX${}_{2}$ (X = H, Me, Et). A common value scale (in au) is used: −0.02—red, −0.01—yellow, 0.00—green, 0.01—cyan, 0.02—blue. The values given are the maximum electrostatic potential (in au).

It is worth mentioning that ZnH${}_{2}$ differs substantially from both ZnMe${}_{2}$ and ZnEt${}_{2}$ with regard to the properties of the hydrogen atoms. Namely, taking into account electronegativities of Zn (1.7), C (2.5) and H (2.2), only in ZnH${}_{2}$ do the hydrogen atoms have a partial negative charge (−0.172 au). For this reason, ZnH${}_{2}$ may tend to form weak dihydrogen bonds with some hydrogen atoms of the R substituents. On the other hand, positively charged hydrogen atoms from Me (0.012 au) and Et (0.008–0.022 au) groups may readily interact with the π-electron system of the R substituents containing the benzene ring. It can also be expected that in some cases, the hydrogen atom of the R substituent can interact with the carbon atom of the X group (especially Me) to form a weak H⋯C interaction. The electrophilic and nucleophilic regions of the ZnX${}_{2}$ molecules can be visually represented by maps of the total electrostatic potential (ESP) projected onto the electron density isosurface (see Figure 4).

It is clearly visible that ZnH${}_{2}$ is distinguished by areas of strongly negative potential located on both hydrogen atoms. In the case of ZnMe${}_{2}$ and ZnEt${}_{2}$, the ESP distributions are similar to each other, although in the former molecule there is a small negative potential region near the center of the side surface of the methyl groups. Importantly, the zinc atom in ZnH${}_{2}$ has a higher potential value (0.049 au) than in ZnMe${}_{2}$ and ZnEt${}_{2}$ (0.036 au and 0.037 au, respectively). This is in line with weak +I effects of alkyl groups.

Despite all these possibilities, however, some general conclusions can be found. Namely, it can be seen that the complexes involving ZnH${}_{2}$ are characterized by a shorter C⋯Zn bond (2.118–2.149 Å) than those involving ZnMe${}_{2}$ (2.170–2.198 Å). The complexes with ZnEt${}_{2}$, on the other hand, are in the middle, and their range (2.137–2.169 Å) overlaps with that for ZnH${}_{2}$. Thus, in terms of the C⋯Zn length, the complexes with ZnEt${}_{2}$ are much more similar to those with ZnH${}_{2}$ than with ZnMe${}_{2}$. This result can be explained as follows. First, a small ZnH${}_{2}$ molecule can approach the carbene closer than ZnMe${}_{2}$ or ZnEt${}_{2}$, having larger groups and therefore sterically interacting with more bulky R substituents (e.g., Ad, Dipp, Mes). Secondly, as already mentioned, the hydridic hydrogen atom of ZnH${}_{2}$ can form weak dihydrogen bonds, as in the complex with I, i.e., imidazol-2-ylidene (see at the top of Figure 2). In turn, the longer aliphatic chain in ZnEt${}_{2}$ (compared to ZnMe${}_{2}$) allows for more favorable attractive interactions (e.g., of the π⋯H-C type) with large R groups of the carbene unit.

It is worth emphasizing that the characteristic effect associated with the formation of the zinc bond is a significant bend of the ZnX${}_{2}$ molecule (more precisely, the E-Zn-E angle where E is the atom directly bonded to Zn). This effect has previously been described extensively [64,84] and is analogous to the bending effect of the BeX${}_{2}$ [64,85,86,87,88,89,90] or MgX${}_{2}$ [64,91,92] molecule during beryllium and magnesium bond formation, respectively. The respective values of the $\alpha_{\mathrm{EZnE}}$ angles are shown in Table 3.

The greatest effect occurs in the complexes of IAd (by far the greatest bend of ca. 128${}^{\circ }$ occurs in IAd–ZnEt${}_{2}$). This result cannot be due solely to the large size of the adamantyl group, as in the case of much smaller methyl groups in IMe, the angle $\alpha_{\mathrm{EZnE}}$ does not differ significantly, especially when X = Me (137.7${}^{\circ }$ vs. 136.5${}^{\circ }$ for IMe and IAd, respectively). On the other hand, the smallest bend of E-Zn-E (151.1${}^{\circ }$) takes place in the IDipp–ZnEt${}_{2}$ complex. For a given X, the I-ZnX${}_{2}$ complexes also exhibit a relatively weak bending effect of E-Zn-E. It can be easily noticed that, with the exception of IMe and IDipp, for a given carbene, the E-Zn-E bending effect is weakest for X = Me and by far strongest for X = Et, which most likely results from considerable steric interactions R⋯Et. The angle $\alpha_{\mathrm{EZnE}}$ does not correlate with the distance C⋯Zn or the dissociation energy.

It has been shown [64] that another structural effect that occurs during the formation of the carbene–ZnX${}_{2}$ complex is the opening of the N-C-N angle ($\alpha_{\mathrm{NCN}}$) in the carbene. The results shown in Table 4 confirm this. However, the variation in $\alpha_{\mathrm{NCN}}$ (the range of $\Delta \alpha_{\mathrm{NCN}}$ amounts to 1.4${}^{\circ }$–2.7${}^{\circ }$) is not large due to significant ring stiffness of the imidazol-2-ylidene unit.

For a given carbene, ZnH${}_{2}$ most often leads to the greatest opening effect. This effect is also slightly greater for ZnEt${}_{2}$ than for ZnMe${}_{2}$. Again, $\Delta \alpha_{\mathrm{NCN}}$ does not correlate well with either the C⋯Zn distance or the dissociation energy. It therefore seems that small changes occurring in the ring of the imidazol-2-ylidene unit are due to various subtle effects.

As mentioned earlier, the angle between the plane of the ZnX${}_{2}$ molecule (more precisely its E-Zn-E fragment) and the plane of the carbene (its imidazol-2-ylidene ring) depends largely on the size of the R substituent in the carbene molecule. This issue will now be discussed in more detail. The relevant values of the torsional angle are given in Table 5.

In the case of the smallest imidazol-2-ylidene, the I–ZnX${}_{2}$ complex is flat (see also the front view in the top row of Figure 2). In the case of ZnH${}_{2}$, such a complex structure may be partially due to the presence of a dihydrogen bond N-H⋯H-Zn, while in the case of ZnMe${}_{2}$ and ZnEt${}_{2}$, it may be due to probably slightly stabilizing N-H⋯C interactions (see top view in Figure 2). In the case of a somewhat larger IMe, the ZnX${}_{2}$ plane starts to twist slightly when X is an ethyl group (10.7${}^{\circ }$) and, in particular, a methyl group (28.7${}^{\circ }$). The explanation should most likely be sought in the most favorable balances between supposedly destabilizing H⋯H interactions and stabilizing C-H⋯C interactions at such θ angles. The torsion angle discussed is clearly greater (ca. 42${}^{\circ }$–45${}^{\circ }$) for the I${}^{i}$Pr–ZnX${}_{2}$ complexes, i.e., those having much more bulky isopropyl groups. If even slightly bulkier *tert*-butyl groups are present, then the ZnX${}_{2}$ unit is positioned perpendicularly or nearly perpendicularly (ZnEt${}_{2}$) to the plane of the imidazol-2-ylidene ring.

The presence of the phenyl ring in the carbene substituent R introduces new possibilities of interactions. Namely, the π⋯H-C interactions are possible, although they should be weak due to relatively long distances to the planes of the phenyl rings. Despite the large size of the phenyl groups, they give less steric hindrance than the *tert*-butyl groups, which facilitates the optimization of Ph⋯ZnX${}_{2}$ interactions by appropriately twisting the phenyl groups relative to the plane of the imidazol-2-ylidene ring. This twist is partially forced by a collision interaction between the hydrogen atoms at the positions 4 and 5 of the substituted imidazol-2-ylidene and at the position 2 of the phenyl rings. In the case of ZnMe${}_{2}$ and ZnEt${}_{2}$, the torsion angle of the ZnX${}_{2}$ plane is practically the same and amounts to ca. 67${}^{\circ }$. However, in the case of ZnH${}_{2}$, it is significantly greater and amounts to ca. 83${}^{\circ }$. In combination with the torsion of the phenyl rings, such arrangement of ZnMe${}_{2}$ and ZnEt${}_{2}$ units enables long-range π⋯H-C intermolecular interactions (see Figure 2). In contrast, in the case of ZnH${}_{2}$, the π-electron system of the phenyl groups should repel the negative hydrogens of the ZnH${}_{2}$ molecule (Figure 2) leading to a greater twist, which is actually the case.

In the case of single, double, and triple methylated phenyl groups, i.e., at the transition to ITol, IXyl, and IMes, the ZnH${}_{2}$ unit is perpendicular, which probably results from a slightly greater (as a result of the +I effect of Me) unfavorable effect of π⋯H-Zn. On the other hand, in the case of ZnMe${}_{2}$ and ZnEt${}_{2}$ molecules, a considerable variation in the values of the θ angle is observed. For a given X, being either Me or Et, the torsional angle for the Xyl and Mes groups is practically the same (ca. 40${}^{\circ }$ and 64${}^{\circ }$, respectively). The greater angle for X = Et should most likely be attributed to the greater steric interactions than for X = Me, which are largely derived from the methyl groups at the 2 and 6 positions of the phenyl rings. In addition, note (Figure 3) that the rings of Xyl and Mes are nearly perpendicular to the plane of the imidazol-2-ylidene unit, particularly for X = Et. In the case of ZnMe${}_{2}$, the change of the R substituent from Ph to Tol, i.e., the substitution of the methyl group in position 4, practically does not affect the torsional angle (change from 66.7${}^{\circ }$ to 66.1${}^{\circ }$), while such a replacement has a small impact in the case of ZnEt${}_{2}$ (change from 66.6${}^{\circ }$ to 59.7${}^{\circ }$). Due to the large distance of the methyl group in Tol to X, these changes should not be ascribed to significantly altered steric effects and are rather due to the difference in subtle electronic effects.

Compared to the Ph, Tol, Xyl, and Mes groups, the Dipp group introduces new possibilities. Namely, on the one hand, a large spatial hindrance and, on the other hand, considerable flexibility in the arrangement of the isopropyl groups allow quite large interactions with the ZnX${}_{2}$ unit. In addition, the faces of the phenyl rings are largely obscured by these groups. As a result, the observed twist angle will be the result of subtle Dipp⋯X interactions, not only unfavorable steric, but also some favorable ones, i.e., locally stabilizing. In the case of ZnH${}_{2}$ and ZnEt${}_{2}$, the angle is similar (54.7${}^{\circ }$ and 53.1${}^{\circ }$ respectively), while it is clearly different (58.3${}^{\circ }$) in the case of ZnMe${}_{2}$. This may result from both the less symmetrical arrangement of the phenyl rings of the Dipp groups (Figure 3) and the relatively strong C-H⋯C interactions, which can be expected considering the distribution of ESP around methyl groups (Figure 4). The adamantyl group is undoubtedly the largest spatially; therefore, it should lead to the greatest steric effects on ZnX${}_{2}$. Indeed, the twist angles for both ZnMe${}_{2}$ and especially ZnEt${}_{2}$ are considerably large (83.7${}^{\circ }$ and 88.0${}^{\circ }$, respectively). What may seem surprising at first is the relatively small twist angle for IAd–ZnH${}_{2}$, which amounts to 68.5${}^{\circ }$ only. However, Figure 3 suggests that this may be due to the presence of two dihydrogen bonds of the C-H⋯H-Zn type, although their length is as large as 2.328 Å. Additionally, such a deviation (θ = 68.5${}^{\circ }$) from the perpendicularly oriented ZnH${}_{2}$ molecule allows for the presence of two C-H⋯Zn interactions (2.264 Å), which in principle can be called (δ)-agostic bonds [93,94,95,96,97,98,99,100,101].

These latter two examples, i.e., the complexes IDipp–ZnX${}_{2}$ and IAd–ZnX${}_{2}$, show that the torsional angle of the ZnX${}_{2}$ unit plane with respect to the plane of the imidazol-2-ylidene ring may depend not only on steric effects, but also on the possibility of obtaining favorable long-range interactions, e.g., of the C-H⋯C, C-H⋯H-C, or C-H⋯Zn type. To my knowledge, although in the context of NHC-M systems, the steric effects derived from substituents in the 1 and 3 positions of imidazol-2-ylidene are often described, the significant influence of possible stabilizing interactions has not previously been adequately addressed.

### 3.3. QTAIM-Based Analysis

#### 3.3.1. Electron Density and Delocalization Index

Undoubtedly, QTAIM [72,73,74] is one of the most frequently used theoretical methods in the description of various types of intra- and especially intermolecular interactions. In the description of the strength of any A⋯B interaction, particularly useful are the electron density computed at the bond critical point of this interaction ($\rho_{A\cdots B}$) and the delocalization index determined for atoms (more precisely, atomic basins) A and B, i.e., δ(A,B). The values of the former parameter determined for the bond critical point of the C⋯Zn zinc bond are presented in Table 6.

Considering the electron density $\rho_{C\cdots \mathrm{Zn}}$ as a measure of the C⋯Zn bond strength, the data presented in Table 6 show that the strongest C⋯Zn contact should be present in the IMes–ZnH${}_{2}$ complex. In contrast, the weakest C⋯Zn should be in IPh–ZnMe${}_{2}$ ($\rho_{C\cdots \mathrm{Zn}}$ amounts to 0.076 au and 0.064 au, respectively). Importantly, for a given carbene, complexes with ZnH${}_{2}$ are characterized by the greatest value of the electron density, whereas their ZnMe${}_{2}$-bonded analogs are characterized by the lowest. Thus, still treating $\rho_{C\cdots \mathrm{Zn}}$ as a measure of the C⋯Zn bond strength, the substitution of both hydrogen atoms in ZnH${}_{2}$ by ethyl groups and especially by methyl groups should weaken this bond. Although the complexes with bulky R and X being either Et or H have somewhat greater $\rho_{C\cdots \mathrm{Zn}}$ values, this is not necessarily the case when X = Me.

**Figure 5:**
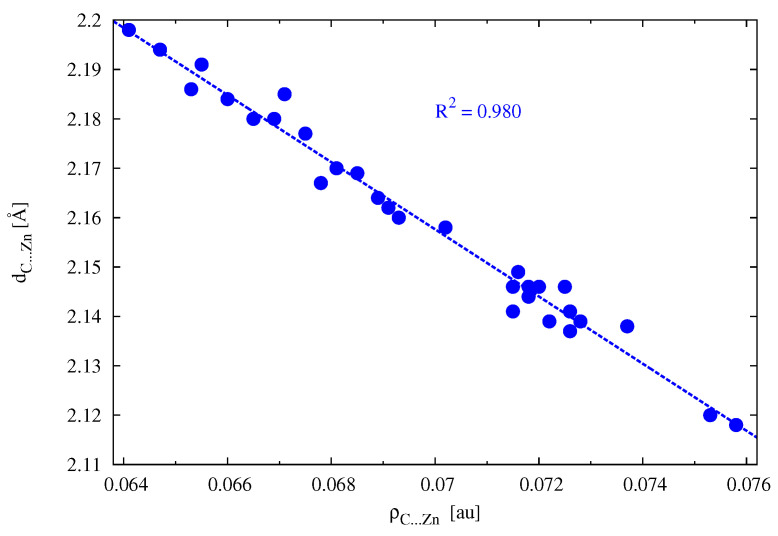
Relationship between the length of the C⋯Zn zinc bond and the electron density determined at the critical point of this bond.

It has been shown previously [64] that for several carbene/CDP–ZnX${}_{2}$ complexes (carbene = cyclopropenylidene, imidazol-2-ylidene; CDP = (PH${}_{3}$)${}_{2}$C or (NH${}_{3}$)${}_{2}$C; X = Br, H), in which interactions other than C⋯Zn are rather insignificant, there was very good ($R^{2}$ = 0.950) linear correlation between $\rho_{C\cdots \mathrm{Zn}}$ and $d_{C\cdots \mathrm{Zn}}$. Figure 5 shows that there is an even better ($R^{2}$ = 0.980) relationship of this type for the complexes considered here. Therefore, a greater value of $\rho_{C\cdots \mathrm{Zn}}$ means a shorter C⋯Zn contact.

As already mentioned, another useful parameter for describing strength of a bond (or an interaction) is the delocalization index [102]. Its values determined for the C⋯Zn bond are presented in Table 7.

In full agreement with the result based on $\rho_{C\cdots \mathrm{Zn}}$ (Table 6), the values of δ(C,Zn) suggest that the strongest C⋯Zn bond should exist in IMes–ZnH${}_{2}$ (0.495) and the weakest in IPh–ZnMe${}_{2}$ (0.405) (and in I${}^{t}$Bu–ZnMe${}_{2}$ for which δ(C,Zn) = 0.406). Like $\rho_{C\cdots \mathrm{Zn}}$ before, the values of δ(C,Zn) also show that for a given carbene, the C⋯Zn bond should be the strongest for ZnH${}_{2}$ and the weakest for ZnMe${}_{2}$.

#### 3.3.2. Molecular Graphs

**Figure 6:**
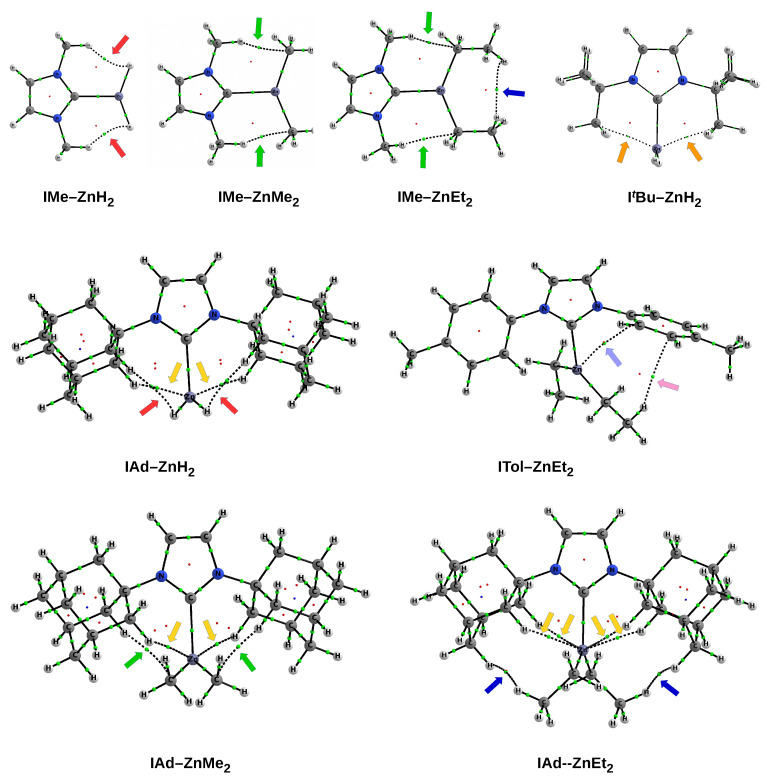
Molecular graphs of selected IR–ZnX${}_{2}$ complexes. Color-coded arrows show the following types of interactions: C-H⋯H-Zn dihydrogen bond (red), C-H⋯C tetrel bond (green), C-H⋯H-C interaction (blue), C-H⋯Zn and C⋯Zn δ-agostic bond (yellow and orange), C-H⋯π hydrogen bond (pink), and Zn⋯π(C) contact (purple).

When discussing the results based on QTAIM, it is worth mentioning that in addition to structural symptoms, the presence of the previously discussed interactions accompanying the leading zinc bond is also suggested by the presence of appropriate bond paths on molecular graphs. Some of the more interesting and representative examples are shown in Figure 6.

As can clearly be seen, in the case of the IMe–ZnH${}_{2}$ complex, two bond paths are present that suggest C-H⋯H-Zn dihydrogen bonds. In contrast, in IMe–ZnMe${}_{2}$, the corresponding bond paths suggest the presence of C-H⋯C tetrel bonds. A similar situation also takes place in a somewhat similar IMe–ZnEt${}_{2}$, but there is also a bond path for the C-H⋯H-C dihydrogen interaction between the two hydrogen atoms of the methyl groups in ethyl groups. The ITol–ZnEt${}_{2}$ complex, in turn, features two bond paths to the π-electron system of one of the Tol groups; one of them is of the C-H⋯π type, the other Zn⋯π. Some complexes feature bond paths that suggest the presence of δ-agostic bonds of the (C-H)⋯Zn type. For example, two C⋯Zn bond paths are present in I${}^{t}$Bu–ZnH${}_{2}$. Complexes IAd-ZnH${}_{2}$ and IAd-ZnMe${}_{2}$ feature two bond paths of the C-H⋯Zn type rather than of C⋯Zn, while IAd-ZnEt${}_{2}$ has as many as four C-H⋯Zn bond paths. Additionally, in the complexes of IAd, there are sequentially two bond paths for the C-H⋯H-Zn dihydrogen bonds, C-H⋯C tetrel bonds, and C-H⋯H-C dihydrogen interactions.

**Figure 7:**
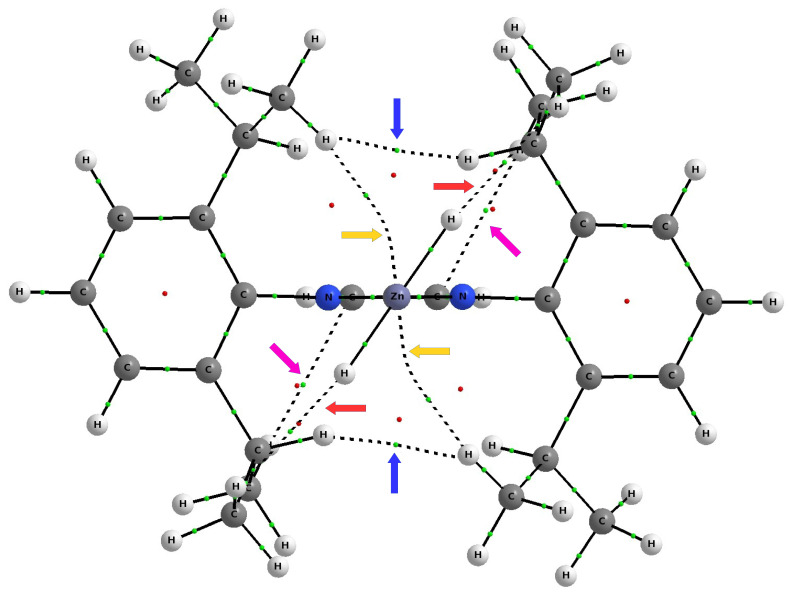
Molecular graph of the IDipp–ZnX${}_{2}$ complex. Color-coded arrows show the following types of interactions: C-H⋯H-Zn dihydrogen bond (red), C-H⋯H-C interaction (blue), C-H⋯Zn contact (yellow), and C-H⋯π hydrogen bond (pink).

It is not unusual that molecular graphs suggest many different long-range interactions through the presence of appropriate bond paths. This is especially true in the case of larger molecular systems with a high concentration of functional groups. A good and quite clear example is the molecular graph of the IDipp–ZnH${}_{2}$ complex shown in Figure 7. On the basis of this molecular graph, it should be concluded that the IDipp–ZnH${}_{2}$ complex simultaneously features two dihydrogen bonds of the C-H⋯H-Zn type, two dihydrogen interactions of the C-H⋯H-C type, two C-H⋯Zn interactions, and two hydrogen bonds of the CH⋯π type (bond paths to the carbon atoms of the imidazol-2-ylidene ring).

As one can see, the IR–ZnX${}_{2}$ complexes considered here present a fairly wide spectrum of various interactions. Their role is difficult to assess. Admittedly, in many cases, the presence of appropriate bond paths may suggest the most important interatomic contacts, but on the other hand, it has been shown [90,103,104] that bond paths often do not show dominant interactions and, importantly, generally have little to do with the energy of a given interaction. Nevertheless, this analysis, together with the analysis based on geometrical parameters presented earlier, strongly suggests that the structure of the IR–ZnX${}_{2}$ complexes (and also more generally NHC–M) depends not only on the steric interactions between the R substituents in imidazol-2-ylidene and X groups in the attached ZnX${}_{2}$ unit, but may also be conditioned by the presence of various interactions between the atoms of R and ZnX${}_{2}$. At least some of them may be locally attractive, i.e., stabilizing, in nature.

### 3.4. NCI-Based Analysis

**Figure 8:**
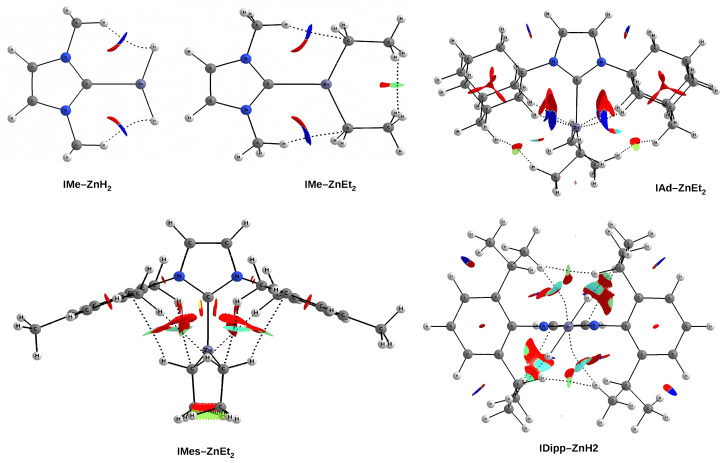
Reduced gradient density isosurfaces (s = 0.3 au) for some representative IR–ZnX${}_{2}$ complexes. Colors are coded according to a common sgn($\lambda_{2}$)ρ scale (in au): −0.008—blue, −0.006—cyan, −0.004—green, −0.002—yellow, 0.000—red. A cutoff of 0.030 au was used for the electron density.

Parameters based on QTAIM are often local as they relate to specific critical points on the molecular graph and are in a way blind to what is happening in the immediate vicinity of these points. One way out of this limitation is the NCI method [75,76], which is based on the value of the reduced electron density gradient, $s=1/\left(2{\left(3\pi^{2}\right)}^{1/3}\right)\left|\nabla \rho \right|/\rho^{4/3}$. This method allows one to isolate and then illustrate weak interactions by applying appropriate cutoffs on the values of both the electron density and its reduced gradient. Then, the individual weak interactions are displayed as certain regions of real space rather than simply as a bond critical point between a pair of atoms [75]. To further investigate the nature of the weak interactions existing between the R substituents in imidazol-2-ylidene and the ZnX${}_{2}$ moiety, these interactions were ‘isolated’ by using a fairly low electron density cutoff of 0.030 au. (Note that the C⋯Zn bond considered earlier is characterized by much higher values in the range of 0.064–0.076 au; Table 6.) To further increase the sensitivity of distinguishing the strength of the respective weak interactions within the NCI method [75], a very narrow scale of sgn($\lambda_{2}$)ρ values was used, from −0.008 au (blue) to zero (red), so that all (weak) repulsive interactions are shown in red. On the contrary, all the other colors are concerned with weak attractive interactions. Representative examples of NCI plots are shown in Figure 8.

As already mentioned (Figure 6), the IMe–ZnH${}_{2}$ and IMe–ZnEt${}_{2}$ complexes are characterized by the presence of a pair of bond paths for C-H⋯H-Zn dihydrogen bond and C-H⋯C tetrel bond, respectively. These paths pierce the blue portions of the *s* isosurface, suggesting that the interactions are relatively strong (although they obviously belong to weak non-covalent interactions). In the case of the latter of these complexes, it is clearly visible that the C-H⋯H-C dihydrogen interaction should be much weaker, but also stabilizing (green). The case of the IAd–ZnEt${}_{2}$ complex shows that despite the presence of distinct areas of repulsion, the agostic C-H⋯Zn bonds are attractive and stronger than the C-H⋯H-C contacts that indicate the interaction between the Ad and Et groups. A similar situation also takes place in IMes–ZnEt${}_{2}$, although the attractive interactions to the zinc and the C-H⋯π hydrogen bonds should be weaker. It is worth taking a closer look at the interaction zone between the ethyl groups of ZnEt${}_{2}$. Namely, this zone presents two sub-areas with opposite characteristics due to the nature of the interaction. The C⋯C interaction is clearly repulsive, while the interaction between the hydrogen atoms themselves is surprisingly (very weakly) attractive (greenish-yellow).

As discussed earlier (see Figure 7), the complex IDipp–ZnH${}_{2}$ exposes four pairs of bond paths for various types of weak non-covalent interactions (C-H⋯π, C-H⋯H-C, C-H⋯Zn, C-H⋯H-Zn). Their presence causes, in addition to the pronounced areas of repulsion, weakly binding regions to also be present, which again shows that the structure of the IR–ZnX${}_{2}$ (and more generally NCH–M) complexes not only depends on the steric repulsion of R⋯X, but may also to some extent depend on various binding interactions. Depending on the possibility of their formation, dihydrogen bonds, tetrel bonds, and agostic bonds should play a particular role here. Even if these individual interactions are weak, their overall binding effect may be non-negligible.

## 4. Conclusions

This article discusses the results of research on the properties of as many as 30 complexes of the IR–ZnX${}_{2}$ (X = H, Me, Et) type, where IR denotes the imidazol-2-ylidene molecule in which the hydrogen atoms in positions 1 and 3 are replaced by R substituents (Me, ${}^{i}$Pr, ${}^{t}$Bu, Ph, Tol, Xyl, Mes, Dipp, Ad). The main emphasis was placed on the relationship between the type and size of the R substituents and the strength of the C⋯Zn zinc bond. The other issue of paramount importance was the influence of the size and type of R and X on the torsional angle between the ZnX${}_{2}$ plane and the imidazol-2-ylidene ring plane.

It has been shown that the strongest C⋯Zn bond should be present in IMes–ZnH${}_{2}$, and the weakest in IPh–ZnMe${}_{2}$. These results are in full agreement with the highest and lowest electron density and delocalization index computed for the corresponding C⋯Zn bonds. Surprisingly, despite the considerable diversity of the R and X substituents, the distance range C⋯Zn turned out to be quite narrow, from 2.12 Å to 2.20 Å. On the contrary, the dissociation energies are characterized by a fairly wide range, from 18.5 kcal/mol to 27.4 kcal/mol. In general, the IR–ZnH${}_{2}$ complexes should be stronger than their ZnMe${}_{2}$-bonded counterparts, which is also manifested in stronger structural changes in the former case.

It has been shown that the twist angle of the ZnX${}_{2}$ plane with respect to the ring plane of the imidazole-2-ylidene unit strongly depends not only on the size of the R substituent, but also on the type of the X group. In the case of I and IMe, i.e., the smallest carbenes, the ZnX${}_{2}$ molecule is either in the plane of the carbene unit or is only slightly twisted with respect to it. The torsional angle becomes larger and more varied with the bulkier R. However, it is not easy to predict its value ad hoc, because the value of this angle results not only from the presence of steric effects, but also from the possible presence of interatomic interactions, such as the C-H⋯H-Zn dihydrogen bond, the C-H⋯C tetrel bond, the δ-agostic bond, and possibly the C-H⋯π hydrogen bond and the Zn⋯π contact. It has been shown that at least some of these interactions may have a non-negligible influence on the structure of the IR–ZnX${}_{2}$ complex, in particular the twisting angle of the ZnX${}_{2}$ unit with respect to the imidazol-2-ylidene ring. This fact should be taken into account in addition to the commonly discussed R⋯X steric repulsion.

## Figures and Tables

**Table 1:** The length (in Å) of the C⋯Zn zinc bond in the IR–ZnX${}_{2}$ complexes.

X	I	IMe	I${}^{i}$Pr	I${}^{t}$Bu	IPh	ITol	IXyl	IMes	IDipp	IAd
H	2.141	2.146	2.141	2.149	2.144	2.146	2.120	2.118	2.139	2.138
Me	2.186	2.180	2.177	2.191	2.198	2.194	2.184	2.180	2.170	2.185
Et	2.167	2.169	2.158	2.146	2.162	2.160	2.139	2.137	2.164	2.146

**Table 2:** The dissociation energy (in kcal/mol) of the C⋯Zn zinc bond in the IR–ZnX${}_{2}$ complexes.

X	I	IMe	I${}^{i}$Pr	I${}^{t}$Bu	IPh	ITol	IXyl	IMes	IDipp	IAd
H	23.8	25.3	25.3	24.2	22.9	23.6	26.9	27.4	26.2	26.6
Me	18.7	19.1	20.4	19.5	18.5	19.1	23.4	23.8	22.3	21.7
Et	19.9	21.1	22.5	22.5	20.3	21.2	25.2	25.7	24.6	25.7

**Table 3:** The value of the E-Zn-E angle ($\alpha_{\mathrm{EZnE}}$) in the IR–ZnX${}_{2}$ complexes.

X	I	IMe	I${}^{i}$Pr	I${}^{t}$Bu	IPh	ITol	IXyl	IMes	IDipp	IAd
H	145.6	139.3	139.2	138.6	141.0	140.6	141.7	141.3	141.2	135.7
Me	149.9	137.7	139.7	144.7	149.4	148.9	147.0	146.4	146.2	136.5
Et	142.1	132.1	134.0	134.2	140.8	139.3	136.6	136.4	151.1	127.9

**Table 4:** Change of the N-C-N angle ($\Delta \alpha_{\mathrm{NCN}}$) during formation of the IR–ZnX${}_{2}$ complex.

X	I	IMe	I${}^{i}$Pr	I${}^{t}$Bu	IPh	ITol	IXyl	IMes	IDipp	IAd
H	2.4	2.0	2.0	1.6	1.9	1.9	2.7	2.7	2.4	1.8
Me	2.2	1.8	2.0	1.4	1.6	1.7	2.3	2.3	2.5	1.5
Et	2.2	1.8	2.1	2.0	1.8	1.8	2.5	2.5	2.5	1.8

**Table 5:** The torsional angle (θ) of the ZnX${}_{2}$ plane relative to the plane of the imidazol-2-ylidene ring in the carbene.

X	I	IMe	I${}^{i}$Pr	I${}^{t}$Bu	IPh	ITol	IXyl	IMes	IDipp	IAd
H	0.0	0.0	44.7	90.0	82.7	90.0	90.0	90.0	54.7	68.5
Me	0.0	28.7	43.9	90.0	66.7	66.1	41.0	40.4	58.3	83.7
Et	0.0	10.7	41.8	83.3	66.6	59.7	64.4	64.1	53.1	88.0

**Table 6:** Values (in au) of the electron density calculated at the bond critical point of the C⋯Zn interaction ($\rho_{C\cdots \mathrm{Zn}}$) in the IR–ZnX${}_{2}$ complexes.

X	I	IMe	I${}^{i}$Pr	I${}^{t}$Bu	IPh	ITol	IXyl	IMes	IDipp	IAd
H	0.072	0.072	0.073	0.072	0.072	0.072	0.075	0.076	0.073	0.074
Me	0.065	0.067	0.068	0.066	0.064	0.065	0.066	0.067	0.068	0.067
Et	0.068	0.069	0.070	0.072	0.069	0.069	0.072	0.073	0.069	0.073

**Table 7:** Values of the delocalization index determined for the C⋯Zn bond (δ(C,Zn)) in the IR–ZnX${}_{2}$ complexes.

X	I	IMe	I${}^{i}$Pr	I${}^{t}$Bu	IPh	ITol	IXyl	IMes	IDipp	IAd
H	0.481	0.491	0.487	0.463	0.473	0.473	0.493	0.495	0.476	0.479
Me	0.427	0.453	0.450	0.406	0.405	0.409	0.421	0.424	0.428	0.431
Et	0.447	0.468	0.469	0.452	0.437	0.440	0.465	0.467	0.429	0.458

## Data Availability

Data is contained within the article.

## Abbreviations

The following abbreviations are used in this manuscript:

NHC	N-heterocyclic carbene
IR	R-substituted imidazol-2-ylidene
Me	methyl group
Et	ethyl group
${}^{i}$Pr	isopropyl group
${}^{t}$Bu	*tert*-butyl group
Ph	phenyl group
Tol	4-methylphenyl (toluene) group
Xyl	2,6-dimethylphenyl (xylene) group
Mes	mesityl group
Dipp	2,6-diisopropylphenyl group
Ad	adamantyl group
ESP	electrostatic potential
QTAIM	quantum theory of atoms in molecules
NCI	non-covalent interaction (index/method)
